# A loss of FUS/TLS function leads to impaired cellular proliferation

**DOI:** 10.1038/cddis.2014.508

**Published:** 2014-12-11

**Authors:** C L Ward, K J Boggio, B N Johnson, J B Boyd, S Douthwright, S A Shaffer, J E Landers, M A Glicksman, D A Bosco

**Affiliations:** 1 Department of Neurology, University of Massachusetts Medical School, Albert Sherman Center, Worcester, MA, USA; 2 Laboratory for Drug Discovery in Neurodegeneration, Harvard NeuroDiscovery Center, Brigham and Women's Hospital, Harvard Medical School, Cambridge, MA, USA; 3 Department of Cell and Developmental Biology, University of Massachusetts Medical School, Worcester, MA, USA; 4 Department of Biochemistry and Molecular Pharmacology, University of Massachusetts Medical School, Worcester, MA, USA; 5 Proteomics and Mass Spectrometry Facility, University of Massachusetts Medical School, Shrewsbury, MA, USA

## Abstract

Fused in sarcoma/translocated in liposarcoma (FUS/TLS or FUS) is a multifunctional RNA/DNA-binding protein that is pathologically associated with cancer and neurodegeneration. To gain insight into the vital functions of FUS and how a loss of FUS function impacts cellular homeostasis, FUS expression was reduced in different cellular models through RNA interference. Our results show that a loss of FUS expression severely impairs cellular proliferation and leads to an increase in phosphorylated histone H3, a marker of mitotic arrest. A quantitative proteomics analysis performed on cells undergoing various degrees of FUS knockdown revealed protein expression changes for known RNA targets of FUS, consistent with a loss of FUS function with respect to RNA processing. Proteins that changed in expression as a function of FUS knockdown were associated with multiple processes, some of which influence cell proliferation including cell cycle regulation, cytoskeletal organization, oxidative stress and energy homeostasis. FUS knockdown also correlated with increased expression of the closely related protein EWS (Ewing's sarcoma). We demonstrate that the maladaptive phenotype resulting from FUS knockdown is reversible and can be rescued by re-expression of FUS or partially rescued by the small-molecule rolipram. These results provide insight into the pathways and processes that are regulated by FUS, as well as the cellular consequences for a loss of FUS function.

Fused in sarcoma/translocated in liposarcoma, FUS/TLS (or FUS), is a member of the TET family of proteins that also includes Ewing's sarcoma (EWS) and TATA-binding protein-associated factor 15 (TAF15). TET proteins carry out RNA/DNA-processing activities in the context of diverse cellular functions.^1^ FUS is predominately expressed in the nucleus where it functions in transcription, splicing and DNA damage repair and also shuttles to the cytoplasm, where it has been found in translationally active RNA/protein foci, as well as stress granules formed in response to osmotic stress.^2, 3^

FUS is also associated with several human diseases. FUS was originally discovered in the context of an onco-fusion protein that causes malignant myxoid liposarcoma. The N-terminal transcriptional activation domain of FUS is fused to the transcription factor CHOP, forming FUS-CHOP,^4, 5^ which accounts for >90% of myxoid liposarcoma cases.^6^ Similarly, fusion of FUS with either the transcription factor ERG or FEV has been found in some cases of EWS family tumors^7, 8^ or acute myeloid leukemia,^9, 10^ and fusion with ATF1 and either CREB3 L2 or CREB3 L1 will cause angiomatoid fibrous histiocytoma^11^ and low-grade fibromyxoid sarcoma,^12^ respectively. FUS also has a strong link to neurodegenerative disorders such as amyotrophic lateral sclerosis (ALS),^13, 14^ different subtypes of frontotemporal lobar degeneration^15, 16, 17, 18, 19^ and polyglutamine diseases such as Huntington's disease and spinocerebellar ataxia.^20, 21^ The pathological role of FUS in these disorders has not been elucidated, although the observation that FUS is depleted from the nucleus and/or becomes sequestered into aggregates within neurons and glia during the course of neurodegeneration is consistent with a mechanism involving a loss of FUS function.^15, 22, 23^ A role for a loss of FUS function in the context of essential tremor, an adult-onset movement disorder, has also been proposed.^24, 25, 26^

To study the cellular impact of FUS depletion, we developed cellular models of FUS knockdown and discovered FUS to be critical for homeostasis. Knockdown of FUS in both human embryonic kidney 293T (HEK-293T) and neuronal NSC-34 cells caused a significant defect in cellular proliferation. Importantly, the proliferation defect induced by FUS depletion is reversible, as both re-expression of FUS and treatment with rolipram, a phosphodiesterase-4 inhibitor that suppresses oxidative stress, ameliorated this phenotype. A quantitative proteomics analysis revealed various proteins that changed as a function of FUS knockdown, including some that correspond to known RNA-binding targets of FUS. The proteins and pathways uncovered herein not only define the cellular consequences of FUS depletion, but also serve as potential therapeutic targets for ameliorating adverse phenotypes arising from a loss of FUS function.

## Results

### Cell number and viability directly correlate with FUS protein expression

**Figure 1:**
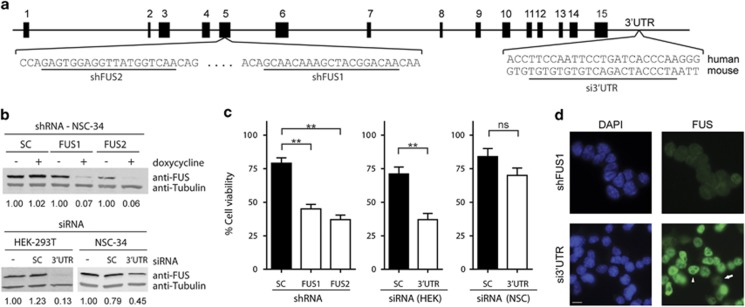
FUS knockdown causes a reduction in cell viability. (**a**) FUS was targeted for knockdown by doxycycline induction of stably expressed shRNA directed at non-overlapping regions in exon 5 (shFUS1, shFUS2) or by transient transfection of siRNA targeting the 3'UTR of either the human or mouse *FUS* sequence. (**b**) Western blot analyses of the indicated cell lysate derived from FUS knockdown experiments. After 4 days of shFUS induction in NSC-34 cells (top) or transient transfection of 3'UTR FUS siRNA in HEK-293T cells (bottom left), FUS expression was reduced by ~95% and ~85%, respectively, compared with controls. A less efficient FUS knockdown of ~55% was observed for NSC-34 cells (bottom right) transiently transfected with 3'UTR FUS siRNA. Numbers below the blots refer to densitometry measurements of the experimental condition (doxycycline-induced or transfected) relative to the untreated control condition for the respective cell line. SC refers to a scrambled (RNA) control sequence. Tubulin serves as a loading control. (**c**) MTT analyses revealed a decrease in cell viability upon FUS knockdown after 4 days compared with SC-expressing cells (left, shRNA in NSC-34 cells; middle, 3'UTR FUS siRNA in HEK-293T cells; right, 3'UTR FUS siRNA in NSC-34 cells). All conditions are reported as percent viability relative to untreated cells. Data shown are the average of three independent experiments±S.E. Statistical significance was determined by a Student's *t*-test (**P<0.01; NS, not significant). (**d**) Representative immunofluorescence images demonstrating the degree of endogenous FUS (green) knockdown in NSC-34 cells induced to express shFUS1 *versus* transiently transfected with 3'UTR FUS siRNA for 4 days. Nuclei are stained with DAPI (blue). Arrow = detectable FUS knockdown, arrowhead = no detectable knockdown. Scale bar = 10 *μ*m

To investigate the cellular consequences of a loss of FUS function, FUS expression was knocked down in both murine NSC-34 (neuroblastoma × spinal cord hybrid 34) and HEK-293T cells. NSC-34 cells are motor neuron-like^27^ and were utilized in light of the involvement of FUS in neurodegeneration,^3^ whereas HEK-293T cells were chosen as a suitable human cell line for *in vitro* experiments. NSC-34 cell lines stably expressed tetracycline-inducible shRNA specific for FUS (shFUS1 and shFUS2; Figure 1a) or a scrambled shRNA control (shSC).^2^ After shFUS induction for 4 days, FUS expression was knocked down ~95% (Figure 1b). In addition, siRNA targeting the 3'UTR of FUS (Figure 1a) or a scrambled siRNA control was used. Transient transfection of 3'UTR siRNA (si3'UTR) for 4 days resulted in ~85% knockdown of FUS in HEK-293T cells (Figure 1b). Cell viability as determined by the MTT assay was reduced 40–50% in NSC-34 cells expressing shFUS and in HEK-293T cells expressing si3′UTR relative to controls (Figure 1c). However, transient transfection of siRNA was less efficient in NSC-34 cells (Figure 1d), resulting in only ~55% FUS knockdown (Figure 1b) and ~15% decrease in cell viability relative to the control (Figure 1c). A reduction in viability (~20%) was also observed in control conditions, likely due to the toxicity associated with transient transfection, continuous shRNA production and/or doxycycline exposure.^28^ Nonetheless, these data demonstrate a reduction in cell viability resulting from FUS knockdown, achieved by targeting several unique sequences within FUS in two different cell lines.

**Figure 2:**
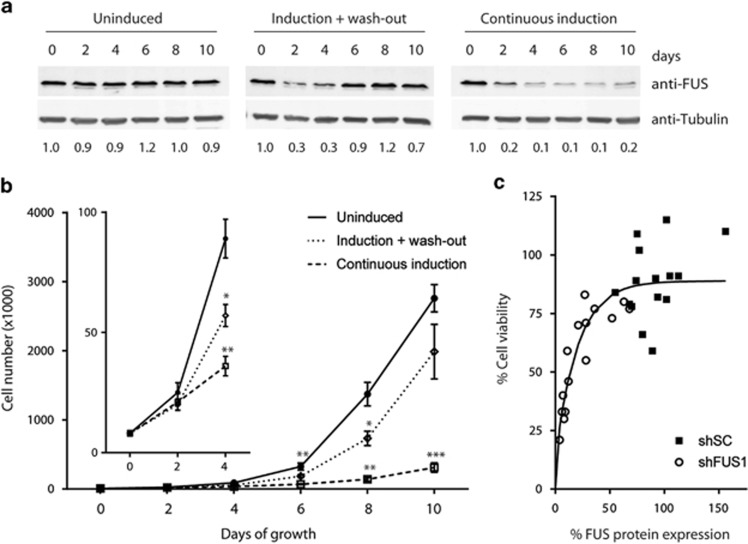
Decreased cell viability correlates with the degree of FUS knockdown. (**a**) NSC-34 cells expressing shFUS1 were uninduced, induced with tetracycline for 24 h after which tetracycline was removed (induction + wash-out) or subjected to continuous induction and FUS knockdown. Western blot analyses of the indicated FUS knockdown experiment reveal FUS expression is restored in the wash-out condition by day 6. Numbers below the blots refer to densitometry measurements that were normalized to ‘day 0', which represents the starting-point of the experiment. Tubulin serves as a loading control. (**b**) Quantification of cell number as a function of time for the indicated conditions. Cells with continuous FUS knockdown exhibited the slowest growth rate between days 2 and 4 (inset) and throughout the 10-day experiment. Upon re-expression of FUS, the growth rate for cells in the wash-out condition was accelerated and became similar to that of the uninduced condition. Data shown are the average of three independent experiments±S.E. Statistical significance was determined by a Student's *t*-test (*P<0.05; **P<0.01; ***P<0.001; day 6 significance is for the continuous induction condition). (**c**) Cell viability, as determined by the MTT assay, correlates with the level of FUS protein expression, as determined by western/densitometry analyses. Data are compiled from three independent FUS knockdown experiments, where shSC or shFUS1 was induced in NSC-34 cells for 1–5 days. An exponential fit of this data was created with GraphPad prism (La Jolla, CA, USA)

To further investigate the relationship between FUS expression and cellular homeostasis, we quantified cell number and viability as a function of FUS depletion over time using the inducible shFUS1 NSC-34 cell line (unless otherwise noted, this line was used for all subsequent experiments). With continuous induction of shFUS, FUS protein levels gradually decreased over time relative to uninduced cells and remained low for 10 days (Figure 2a). Uninduced cells exhibited an exponential growth rate with a 3.5- to 4-fold increase in cell number every 2 days, whereas this growth rate decreased to 2-fold in cells subjected to continuous FUS knockdown (Figure 2b). Plotting cellular viability as a function of FUS expression further demonstrates the direct correlation between FUS expression and cellular homeostasis (Figure 2c). The induction of shFUS was halted after 24 h in a ‘wash-out' experiment, wherein FUS levels returned to baseline within 6 days (Figure 2a). It is noted that tetracycline was used to induce shFUS in these experiments, as the wash-out is ineffective with doxycycline (data not shown). Interestingly, re-expression of FUS rescued the proliferation defect; the rate of cell growth was the same in shFUS cells under the wash-out condition as uninduced cells between days 8 and 10 (Figure 2b). Therefore, the adverse effects of FUS knockdown are reversible.

### Apoptosis is not activated during the cellular response to FUS knockdown

**Figure 3:**
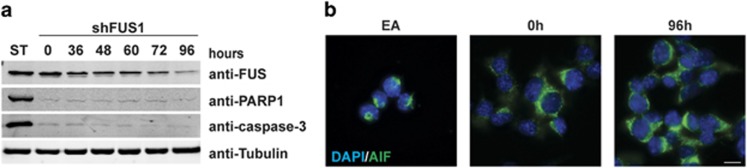
Apoptosis is not activated during the cellular response to FUS knockdown. (**a**) Levels for apoptosis markers, cleaved-PARP1 and cleaved-caspase-3, do not change with FUS knockdown as determined by western blot analyses of cell lysates derived from the indicated time points. As a positive control for apoptosis, NSC-34 cells were treated with 1*μ*M staurosporine (ST) for 2 h. Tubulin serves as a loading control. (**b**) Representative immunofluorescence images demonstrating localization of AIF (green) in NSC-34 cells. AIF translocates to the nucleus in cells treated with 100 *μ*M ethacrinic acid (EA) for 8 h, but not upon shFUS1 induction for 96 h. Images are representative of two independent experiments. Nuclei are stained with DAPI (blue). Scale bar = 10 *μ*m

Several factors influence proliferation rate: the rate of cell division, the percentage of cells undergoing cell division and cell death.^29^ Overt cell death (i.e., a large percentage of cells detaching from the culture dish) was not observed upon FUS knockdown. Western blot analyses confirmed that levels of the apoptosis markers cleaved-PARP1 and cleaved-caspase-3 remained low and unchanged throughout a 96 h FUS knockdown time course (Figure 3a). Moreover, nuclear translocation of apoptosis-inducing factor (AIF) was not observed upon FUS knockdown at 24-h intervals (shown for 96 h in Figure 3b), ruling out that apoptosis occurred through a caspase-independent pathway.^30^

### FUS knockdown results in impaired cell cycle progression

**Figure 4:**
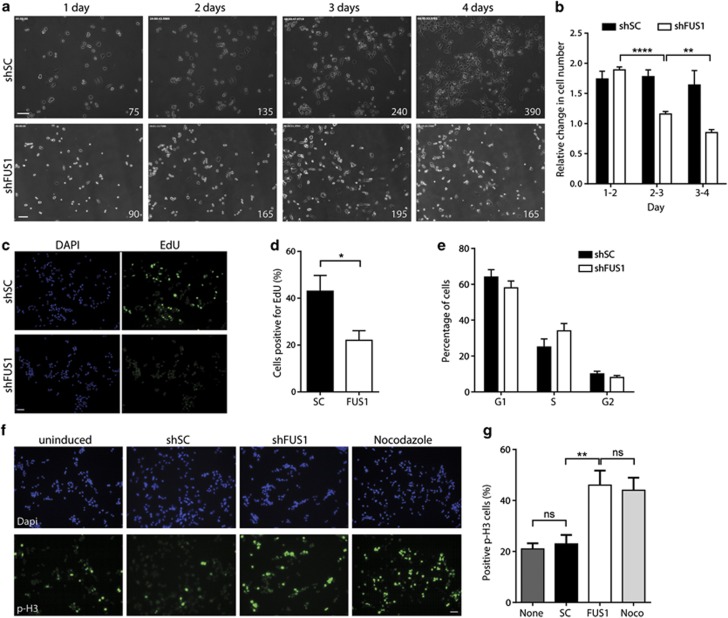
FUS knockdown results in impaired cell cycle progression. (**a**) Still frame images from time-lapse video microscopy obtained every 24h. One day after the induction of shSC (top) or shFUS1 (bottom), cells were filmed using time-lapse microscopy for the duration of a 4-day knockdown. The average number of cells from three independent counts of each image is indicated at the bottom right. Scale bar = 100 *μ*m. (**b**) The change in cell number at 24-h time points relative to the previous time point, where ratios >1.0 are indicative of cell growth and ratios <1.0 of cell death. Data shown are the average of three independent counts of the still frame images + S.D. Statistical significance was determined by a Student's *t*-test (**P<0.01; ****P<0.0001). (**c**) Representative immunofluorescence images showing EdU incorporation (green) after 96 h of either shSC (top) or shFUS1 (bottom) expression. Nuclei are stained with DAPI (blue). Scale bar = 50 *μ*m. (**d**) Quantification of EdU immunofluorescence images revealed a 50% reduction of EdU incorporation for cells expressing shFUS1 for 96 h as compared with cells expressing shSC. Data shown are the average of two independent experiments + S.E. Statistical significance was determined by a Student's *t*-test (*P<0.05). (**e**) Cell cycle analysis by flow cytometry following propidium iodide staining revealed no significant arrest at G1, S or G2 after 96 h of shFUS1 expression in NSC-34 cells. Data shown are the average of seven independent experiments±S.E. (**f**) Representative immunofluorescence images demonstrating p-H3 levels. After 96 h of shFUS1 expression in NSC-34 cells, p-H3 staining (green) is increased to levels comparable to treating cells with 1.6 *μ*M nocodazole for 14 h to induce mitotic arrest. Cells expressing shSC for 96 h are similar to uninduced cells. Nuclei are stained with DAPI (blue). Scale bar = 50 *μ*m. (**g**) Quantification of **f** shows a significant increase in the percentage of p-H3-positive cells upon FUS knockdown or treatment with nocodazole (Noco). Data shown are the average of three independent experiments±S.E. Statistical significance was determined by a one-way ANOVA and Tukey's multiple comparisons test (**P <0.01; NS, not significant)

Time-lapse video microscopy was used to track the fate of a single population of cells during FUS knockdown. Imaging was initiated 24 h after shRNA induction and continued for 3 days. Cell division occurred in shSC cells at a normal frequency (Supplementary Video 1 and Figure 4a), whereas fewer cell division events were observed in shFUS cells (Supplementary Video 2). Quantification of cell number revealed a consistent ~1.7-fold increase in cell number every 24 h for control cells, whereas this change in cell number decreased for shFUS cells (Figure 4b). Moreover, a 50% reduction in EdU incorporation was observed in cells undergoing FUS knockdown for 96 h (Figures 4c and d). Together, these data are consistent with a defect in cell proliferation.

Next, we sought to determine whether a specific stage of the cell cycle was impacted by FUS knockdown. Cell cycle analysis by flow cytometry did not reveal a significant delay in the various stages of the cell cycle (Figure 4e). However, cells undergoing FUS knockdown for 96 h exhibited a robust and significant increase in phosphorylated histone H3 (p-H3) signal as assessed by immunofluorescence microscopy (Figure 4f), indicative of mitotic arrest. In fact, the percentage of cells undergoing FUS knockdown for 96 h that were p-H3-positive (~45%) was comparable to cells treated with nocodazole, an inducer of mitotic arrest (Figure 4g). These data suggest that a defect in mitosis is responsible for the overall reduction in cellular proliferation upon FUS knockdown.

### A phosphodiesterase-4 inhibitor, rolipram, partially rescues the cell proliferation defect induced by FUS knockdown

**Figure 5:**
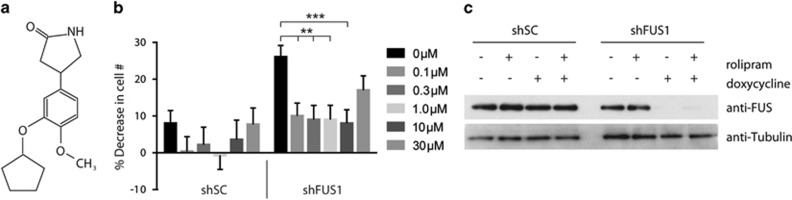
Rolipram partially rescues the cell proliferation defect induced by FUS knockdown. (**a**) Molecular structure of rolipram. (**b**) NSC-34 cells were induced to express shSC or shFUS1 followed by the addition of rolipram at various concentrations (0–30 *μ*M). After 4 days, cell number was quantified relative to uninduced controls by Hoechst staining. In the absence of rolipram, cell number was reduced 25% for shFUS1 and 8% for shSC (*n*= 28 replicate wells from two independent experiments). This defect was significantly reversed with 0.1–10 *μ*M rolipram in shFUS1 cells (*n*= 20 replicate wells from two independent experiments; **P<0.01, ***P<0.001). To a lesser extent, rolipram also recovered the deficit in shSC cells (*n*= 20 replicate wells from two independent experiments). Error bars represent + S.E. Statistical significance was determined by comparing cells (shSC or shFUS1) treated with rolipram to the untreated (0 *μ*M rolipram) condition using the Student's *t*-test. (**c**) Western blot analysis of cell lysates corresponding to the indicated conditions. FUS was knocked down only in cells induced with doxycycline to express shFUS1 and remained knocked down in the presence of 1*μ*M rolipram. Tubulin serves as a loading control

In addition to rescuing the proliferation defect by re-expressing FUS (Figure 2), we sought to determine whether small molecules could exert a similar protective effect. To test this possibility, we screened a chemical library consisting of 1086 small molecules, 606 of which were drugs approved by the Food and Drug Administration (FDA) and 480 of which were purified natural products. Eight compounds were initially identified (0.74% hit rate) that reversed the proliferation defect and five-point dose-response curves in the range of 0.1–30 *μ*M were generated for each. After this analysis, one compound, rolipram (Figure 5a), was found to partially restore the 25% decrease in cell number induced by FUS knockdown (Figure 5b). Rolipram is an inhibitor of cAMP-specific phosphodiesterase-4 with antidepressant and anti-inflammatory functions,^31^ including suppression of nitric oxide production.^32^ The protective effect was less pronounced at 30 *μ*M, suggesting rolipram may exert toxicity at higher doses. Rolipram also increased the cell number deficit caused by shSC expression, however, this effect was modest relative to shFUS1 cells and was not statistically significant (Figure 5b). A western blot analysis demonstrated that the protective effect of rolipram was not because of re-expression of FUS (Figure 5c). These data suggest that rolipram or related compounds could be further explored in the context of therapeutics for disorders arising from a loss of FUS function.

### Proteomic changes resulting from FUS knockdown

**Figure 6:**
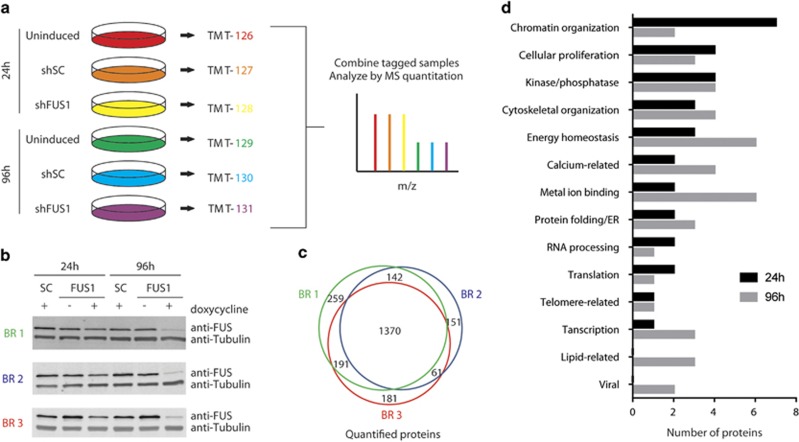
Quantification of the cellular proteome after FUS depletion. (**a**) Schematic of mass spectrometry pipeline. Briefly, NSC-34 cells were induced with doxycycline for 24 or 96 h to express either scrambled control shRNA (shSC) or shRNA targeting FUS (shFUS1). Uninduced cells were included as an additional control. Denatured lysates were digested and the resulting peptides were labeled with unique isobaric mass tags (TMTs 126-131) and combined for mass spectrometry (MS) analysis. (**b**) Western blot analysis confirmed FUS knockdown in lysates used for proteomics. Three biological replicates (BR 1, green; BR 2, blue; BR 3, red) were included. Tubulin serves as a loading control. (**c**) A total of 2355 proteins were quantified by mass spectrometry over three biological replicates (BR 1–3). (**d**) Proteins differentially expressed between the shSC and shFUS1 samples by a fold change of at least ±0.25 in two of three replicates were grouped into functional categories based on the literature (Table 1)

Next, we sought to better understand the pathways involved in the proliferation defect observed upon FUS depletion. To this end, we performed quantitative proteomics using tandem mass tags (TMTs) and mass spectrometry to assess changes in the proteome after 24 and 96 h of FUS knockdown.^33, 34^ This approach allows for multiplexing of multiple conditions within a single experiment and thus an accurate comparison of protein levels between conditions. Briefly, the N-termini of tryptically cleaved peptides are labeled with isobaric mass tags. Each tag adds the same molecular mass, but upon tandem mass spectrometry (MS/MS) analysis, a unique reporter ion is detected for each condition. The resulting reporter ion intensity directly correlates to peptide abundance (Figure 6a). The 24-h time point is expected to reflect an early response to FUS knockdown, when FUS protein expression was reduced by only ~50% (Figure 6b) and cell viability by only ~25% (Figure 2c). The 96-h time point was expected to reveal more robust changes because the FUS knockdown phenotype is more severe (Figures 1c and 6b) and this later time point allows for an accumulation of protein expression changes, which depend on the timescale of both protein translation and turnover. Although it is standard to only compare cells with a specific gene knocked down to cells expressing a scrambled RNA sequence, we also included the uninduced condition in the quantitative proteomics pipeline (Figure 6a) to ascertain whether a particular protein changed in expression solely as a consequence of FUS knockdown, or whether the process of shRNA induction influenced the expression of that protein.

First, changes in the proteome arising from a loss of FUS were assessed by comparing shFUS and shSC samples. A total of 2355 proteins were quantified, with ~60% (1370 proteins) overlap between the three biological replicates (Figure 6c). Only those proteins differentially expressed between the shFUS and shSC samples by a fold change of at least ± 0.25 in two of three replicates with a *P*-value <0.05 were included in our final analysis. Sixty-one proteins, in addition to FUS, met this criterion at either 24 or 96 h (Table 1). Interestingly, 15 of these hits were previously identified as RNA-binding targets of FUS, raising the possibility that these expression changes stem from a loss of FUS function and/or interaction with the corresponding transcripts (Table 1, asterisks).^35, 36, 37^ Of the 61 hits, 31 were various histone variants. These were grouped into five main histone clusters (H1, H2A, H2B, H3 and H4) because of the redundancy of the peptides used to identify each protein. Similarly, KAP0 and KAP1, the regulatory subunits of protein kinase A, were counted as a single hit because of a lack of unique peptides identified for each protein. DAVID analysis (Database for Annotation, Visualization, and Integrated Discovery) could not identify significant enrichment of GO terms or functional categories for the remaining 34 proteins, and therefore hits were assigned categories manually based on information in UniProtKB, the National Center for Biotechnology Information (NCBI) and GeneCards (Figure 6d and Table 1). Chromatin organization represented the most common category for proteins differentially expressed at 24 h. Categories for which there were at least four differentially expressed proteins at either 24 or 96 h included cellular proliferation, kinases/phosphatases, cytoskeletal organization, energy homeostasis, calcium-related and metal ion binding. Of these, energy homeostasis, calcium-related and metal ion binding were more prominent at 96 than 24 h, suggesting these changes are triggered by FUS knockdown and become more pronounced with time.

**Figure 7:**
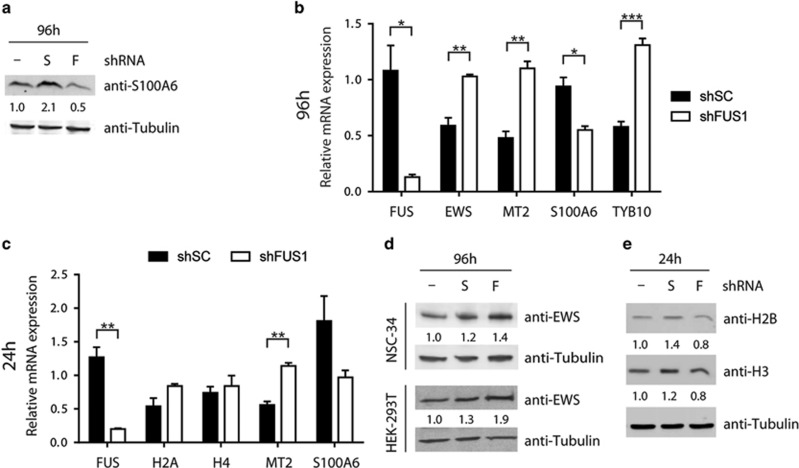
Western and qRT-PCR validation of proteins differentially expressed upon FUS depletion. (**a**) Western blot analysis of lysates from uninduced NSC-34 cells (–) and cells induced to express scrambled control shRNA (S) or shRNA targeting FUS (F) for 96 h. Numbers below the blots refer to densitometry measurements relative to uninduced cells and demonstrate increased expression of S100A6 in shSC cells with a concomitant decrease in shFUS1 cells. Tubulin serves as a loading control. (**b** and **c**) qRT-PCR analysis after 96 h (**b**) or 24 h (**c**) of shRNA induction. RNA expression relative to uninduced cells was determined for the indicated genes after shSC or shFUS1 induction in NSC-34 cells. Data shown are the average of three independent experiments + S.E. Statistical significance was determined by a Student's *t*-test (*P<0.05; **P<0.01; ***P<0.001). (**d**) Representative western blot analysis of lysates from NSC-34 cells or HEK-293T cells after 96 h demonstrating increased expression of EWS with FUS knockdown relative to controls. (**e**) Representative western blot analysis of lysates from NSC-34 cells induced to express shRNA for 24 h demonstrating increased histone expression in shSC cells with a concomitant decrease in shFUS1 cells

Next, we validated protein hits that exhibited the largest expression changes upon FUS knockdown, which were most evident at 96 h. S100A6, also known as calcyclin because of roles in both calcium binding and the cell cycle,^38^ exhibited a relatively large decrease in expression in shFUS cells compared with shSC cells at 96 h (Table 1). By comparing both lines to the uninduced condition, it became apparent that this difference is due in part to an increase in expression of S100A6 in shSC cells (Supplementary Table 1). Differential expression of S100A6 was validated by both western blot (Figure 7a) and qPCR analyses at 96 h (Figure 7b) and qPCR at 24 h (Figure 7c). Metallothionein-2 (MT2), a multifunctional protein involved in zinc homeostasis and antioxidation,^39^ exhibited a large increase in expression in shFUS cells relative to shSC cells at 96 h (Table 1). The differential expression of MT2 in shFUS and shSC cells was validated by qPCR at 96 h (Figure 7b) and 24 h (Figure 7c), however, this protein could not be detected by western blot, presumably because of the relatively small size of MT2 (6 kDa). The RNA-binding protein EWS, which like FUS is a member of the TET family of proteins, also increased in expression in shFUS cells (Table 1,Figures 7b and 7d), as well as in HEK-293T cells transiently transfected with si3'UTR for FUS knockdown (Figure 7d), consistent with a previous report.^40^ This appears to be a specific relationship between FUS and EWS expression as FUS knockdown did not influence the expression of other related RNA-binding proteins such as TDP43 and hnRNPA1, or the other TET family member TAF15 (data not shown). Similar to EWS, thymosin beta-10, a 5 kDa monomeric actin-binding protein that inhibits actin polymerization,^41^ also increased in expression upon FUS knockdown (Table 1 and Figure 7b), but could not be detected by western blot.

Finally, we validated the differential expression of histones, which decreased in expression by 20–65% after 24 h of FUS knockdown but recovered by 96 h (Table 1). This difference arises from an increase in expression of histones in shSC cells relative to both shFUS and uninduced cells (Supplementary Table 1), which was validated by western blot analysis for histones H2B and H3 (Figure 7e). Histones H2A and H4, which were investigated based on availability of PCR primers, were not significantly different between lines by qPCR (Figure 7c) suggesting that the change in histone expression predominately occurs at the protein level.

## Discussion

Genetic alterations in FUS cause human diseases such as cancer and neurodegeneration. In the study of sarcomas caused by fusion of *FUS* with various transcription factors, overexpression of the transcription factor component alone was unable to promote tumor growth,^42^ suggesting that FUS is essential for the oncogenic role of FUS-fusion proteins. A loss of FUS function is implicated in the mechanisms associated with various neurodegenerative disorders, however, the multifunctional nature of FUS has made it difficult to discern which of these functions is relevant to neurodegeneration.^3^ Furthermore, the effect of FUS knockdown or knockout *in vivo* has produced conflicting results, as some reports describe animal lethality,^43, 44, 45, 46^ whereas others report no effect on survival.^47, 48, 49^ Consistent with the latter, signs of apoptosis or gross cellular death were not observed here upon knockdown of FUS in cultured mammalian cells (Figure 3). Rather, we demonstrate for the first time that a reduction in FUS expression impairs cell proliferation and progression through mitosis (Figures 1,2,3,4). Early studies of FUS knockout mice reported a defect in meiosis, specifically within prophase,^48^ a step that has common features in both meiosis and mitosis. This could suggest FUS is required for progression of both mitosis and meiosis through a common role in prophase.

To gain mechanistic insight into the correlation between FUS expression and cell proliferation, we performed quantitative proteomics on cells undergoing various degrees of FUS knockdown, as no other study to date has performed a comprehensive analysis of protein expression changes as a function of FUS depletion. As one may expect, proteins associated with cell proliferation changed in expression upon FUS knockdown (Figure 6d,Table 1). One of these proteins, S100A6, exhibited reduced expression after 96 h (Figure 7, Table 1), consistent with previous reports describing reduced cell proliferation upon S100A6 knockdown.^38, 50^ A role for FUS in cell cycle has been described previously, as FUS inhibits expression of the cell cycle protein cyclin D1 in response to DNA damage signals^51^ and binds RNAs corresponding to cell cycle genes.^35^

MT2 exhibited one of the largest changes in expression between shFUS and shSC cells (Table 1). MT2 has a protective role against metal toxicity, oxidative stress and ionizing radiation. Accordingly, levels of MT2 increase in response to these stressors.^39, 52^ That expression of MT2 was higher in shFUS cells relative to shSC cells at 24 h, a difference that became more pronounced at 96 h (Figure 7,Table 1), implicates a role for one of the aforementioned stressors in the FUS knockdown-induced proliferation defect. We speculate that oxidative stress may be the relevant stressor, as FUS regulates the transcription of oxidative stress protection genes and a loss of this function leads to elevated ROS.^53^ In support of this notion, rolipram partially rescued the proliferation defect upon FUS knockdown (Figure 5). Rolipram has been shown to boost expression of antioxidizing enzymes,^54^ suppress nitric oxide levels^32, 54^ and exert a protective effect in animal models of spinal cord injury^55^ and neurodegeneration.^56, 57, 58^ Although the impact of rolipram on cellular proliferation was strongest for shFUS cells, we note a small effect on the control line that may signify a general effect of rolipram on cell proliferation.^59, 60^ Therefore, rolipram could be further assessed in alternative models for loss of FUS function.

Consistent with a previous report, we also detected an increase in EWS expression upon FUS knockdown (Figure 7,Table 1).^40^ EWS and FUS are both members of the TET family of proteins and exhibit overlapping functions,^1^ raising the intriguing possibility that increased EWS expression serves as a compensatory mechanism for the loss of FUS function in cells. An alternative, although not mutually exclusive, explanation is that regulation of EWS at the RNA level is directly impacted by FUS knockdown. In addition to EWS, 14 other hits correspond to reported RNA-binding targets of FUS (Table 1, asterisks).^35, 36, 37^ We posit that the protein expression changes for these targets are a direct consequence of a loss of FUS function with respect to RNA processing.

In agreement with previous studies, our proteomics analysis revealed protein expression changes induced by doxycycline and/or shRNA expression (i.e., proteins that changed in shSC cells relative to uninduced cells; Supplementary Table 1).^28, 61, 62^ In fact, a recent study reported that expression of genes involved in glycolysis and cellular metabolism were altered upon doxycycline treatment.^28^ We also detected differential expression for proteins associated with energy homeostasis (FPPS, IDHC and KAD1) upon shSC induction at 96 h. Intriguingly, these same changes were not detected in shFUS cells (Supplementary Table 1). Thus, doxycycline exposure and/or shRNA expression elicited changes in gene expression, but under conditions of FUS knockdown this response was impaired. These results underscore the importance of including an untreated control condition to assess the direction of change for the experimental condition; without the untreated condition we would have reported a decrease in histones for shFUS cells at 24 h, when in fact there was an increase in shSC and no response in shFUS cells (Supplementary Table 1). The reason histones are increased in shSC cells at 24 h is unclear, however, the association of FUS with different histone-related processes may explain the lack of response in shFUS cells.^35, 51, 63, 64^

The results of our study have important implications for disease, particularly neurodegenerative disorders that may be mediated through a loss of FUS function. Although mature neurons are post mitotic and do not proliferate, the loss of FUS function may adversely impact neurodevelopment during a stage when cellular proliferation is critical. Moreover, the processes and proteins that are altered here upon FUS knockdown, particularly those relating to cytoskeletal organization, oxidative stress and calcium handling, are relevant to the homeostasis of mature neurons.^65^ We also note that glia cells, which do proliferate, have both neuroprotective and pathogenic roles in neurodegeneration.^66^ That FUS is aggregated and depleted from the nucleus of glia cells in some neurodegenerative disorders^23, 67^ raises the possibility that glial proliferation and function may also be impaired. Importantly, our data demonstrate it is possible to reverse the adverse effects of FUS depletion by replacing the FUS protein or by small-molecule intervention. Proteins that exhibit differential expression upon FUS knockdown (Table 1) may also serve as therapeutic targets for ameliorating the loss of FUS function in disease.

## Materials and Methods

### Tissue culture

The creation of and culture conditions for NSC-34 cell lines expressing shFUS1 or shSC have been described previously.^2^ The tetracycline-inducible shFUS2 line was created and cultured in the same manner (shFUS2: 5′-GAGTGGAGGTTATGGTCAA-3′). For the expression of shRNA, cells were treated with 1 *μ*g/ml doxycycline (Sigma-Aldrich (St. Louis, MI, USA), D9891) or 0.1 *μ*g/ml tetracycline (Fisher Scientific (Loughborough, England), BP912-100). Naive NSC-34 cells (a kind gift from Dr. Neil Cashman) were cultured in Dulbecco's modified Eagle's medium with sodium pyruvate (Invitrogen (Grand Island, NY, USA), 10313) supplemented with 10% fetal bovine serum (Sigma-Aldrich, F4135), 2 mM L-glutamine (Invitrogen, 25030081) and 1% penicillin and streptomycin solution (Invitrogen, 15140122). HEK-293T cells were maintained in minimum essential media (Invitrogen, 10370) supplemented with 10% fetal bovine serum (Sigma-Aldrich, F4135) and 1% penicillin and streptomycin solution (Invitrogen, 15140122).

### siRNA knockdown of FUS

RNA oligonucleotides for FUS (sequences below) and a scrambled control sequence (guide: 5′-AAUUCUCCGAACGUGUCACGU-3′ passenger: 5′-GUGACACGUUCGGAGAAUCUU-3′) were purchased through Sigma-Aldrich and 10 *μ*M annealed stocks were prepared by combining guide and passenger strands in the following buffer: 100 mM potassium acetate, 30mM HEPES-KOH pH 7.4, 2 mM magnesium acetate. For knockdown in HEK-293T cells, cells were plated in a 24-well plate at 2000 cells per well and allowed to adhere overnight. Transfection was performed in OPTI-MEM (Invitrogen, 31985070) using 0.4 *μ*l Lipofectamine RNAiMax (Invitrogen, 13778030) and 0.2 nM siRNA per well (guide: 5′-UUGGGUGAUCAGGAAUUGGAA-3′ passenger: 5′-CCAAUUCCUGAUCACCCACUU-3′). For knockdown in naive NSC-34 cells, cells were plated in a 24-well plate at 10 000 cells per well. Transfection was performed as above, but with 0.5 *μ*l RNAiMax and 10 nM siRNA per well (guide: 5′-UAGGGUAGUCUGACACACACA-3′ passenger: 5′-UGUGUGUCAGACUACCCUCUU-3′).

### MTT viability assay

Cells were plated in technical triplicate in a 24-well plate and induced to express shRNA or transfected with siRNA to initiate FUS knockdown. After the desired duration of knockdown, the media from each well was removed and replaced with 400 *μ*l fresh media and 100 *μ*l of 5 mg/ml MTT (3-[4,5-dimethylthiazol-2-yl]-2,5-diphenyl tetrazolium bromide; Invitrogen, M-6494). After a 35-min incubation at 37 ºC, 300 *μ*l lysis buffer (10% SDS in 1:1 *N*,*N*-dimethylformamide:water/2% acetic acid/2.5% HCl 1M)^68^ was added to each well. Plates were covered with a seal and incubated at 37 °C overnight followed by OD quantification at 550 nm. Results are expressed as % cell viability relative to untreated controls using the following equation: 100 × (OD_RNAi_–OD_blank_)/(OD_untreated_ – OD_blank_). The OD_blank_ was determined by wells containing MTT and tissue culture media without cells.

### Immunofluorescence

Cells grown on glass coverslips were fixed for 5–10 min with 4% paraformaldehyde and blocked with PBSAT (1X phosphate-buffered saline (PBS)/1% BSA/0.5% Triton-X 100) for 30–60 min at room temperature. Primary antibodies were diluted in PBSAT and added to the coverslips at room temperature for 1 h. Primary antibody dilutions were as follows: 1 : 500 for anti-FUS (Bethyl laboratories (Montgomery, TX, USA), A300-293A), 1 : 100 for anti-AIF (Cell Signaling (Danvers, MA, USA), 4642) and 1 : 250 for p-H3 (Santa Cruz Biotechnology (Santa Cruz, CA, USA), sc-8656-R). After primary antibody incubation, coverslips were washed with PBSAT and incubated for 45 min with Alexa Fluor 488-conjugated donkey anti-rabbit IgG (Jackson ImmunoResearch Labs (West Grove, PA USA), 711-545-152), diluted at 1 : 2000 in PBSAT. Finally, cells were stained with 34̀ng/ml DAPI and coverslips were mounted with ProLong Gold anti-fade reagent (Invitrogen, P36930). The percentage of cells positive for p-H3 was determined with MetaMorph V7.6.3 software (Molecular Devices, Sunnyvale, CA, USA) using the Multi Wavelength Cell Scoring application. Briefly, immunofluorescence images were opened in MetaMorph and using the application, cells were identified by DAPI staining and scored for p-H3 based on a 488 nm intensity of 1000 gray levels above background. The number of cells positive for p-H3 was divided by the total number of cells identified by DAPI staining to determine a percentage.

### Western blotting

Cells were lysed for 30 min at 4 °C with either 50 mM Tris HCl (pH 7.5) containing 0.5 M NaCl, 1% NP-40, 1% deoxycholic acid, 0.1% SDS and 2 mM EDTA or RIPA buffer (Boston BioProducts (Ashland, MA, USA), BP-115-500), supplemented with protease (Roche (Basel, Switzerland), 11836170001) and phosphatase (Roche, 4906837001) inhibitors. Concentration of cell lysates was determined using a bicinchoninic acid (BCA) assay (Thermo Scientific Pierce (Rockford, IL, USA), 23227) and samples were prepared in Laemmli SDS-sample buffer (Boston BioProducts, BP-111R). Standard western blotting procedures were followed using Tris-glycine SDS-PAGE and electrotransfer onto PVDF membrane at 100 V for 1 h at 4 ºC. For detection of S100A6, Tris-tricine SDS-PAGE was used (Bio-Rad (Hercules, CA, USA), 456-3066) followed by electrotransfer onto 0.22 *μ*m nitrocellulose at 100 V for 30 min at 4 ºC. Membranes were blocked for 1 h at room temperature with Odyssey Blocking Buffer (LiCor (Lincoln, NE, USA), 927–40003) diluted 1 : 1 with PBS containing 0.1% Tween-20 (PBST), followed by overnight incubation at 4 °C with primary antibodies diluted in PBST. Primary antibody dilutions were as follows: 1:500 anti-FUS (Genscript (Piscataway Township, NJ, USA), generated against C-terminal peptide CKFGGPRDQGSRHDSEQDNSD)^2^, 1:500 anti-tubulin (Sigma (St. Louis, MO, USA), T9026), 1 : 500 anti-cleaved PARP1 (Cell Signaling, 9544), 1 : 500 anti-cleaved caspase-3 (Cell Signaling, 9664), 1 : 500 anti-EWS (Santa Cruz Biotechnology, sc-28327), 1 : 1000 anti-H2B (Active motif, Carlsbad, CA, USA), 1 : 1000 anti-H3 (Abcam (Cambridge, England), 1791) and 1 : 200 anti-S100A6 (Cell Signaling, 13162). Following primary antibody incubation, membranes were washed with PBST and incubated for 1h with secondary antibody diluted 1 : 10 000 in 1:1 Odyssey Blocking Buffer:PBST. Secondary antibodies included anti-mouse IRDye 680 (LiCor, 926-68072) or IRDye 800 (LiCor, 926-32210) and anti-rabbit IRDye 680 (LiCor, 926-68023) or IRDye 800 (LiCor, 926-32211). After washing with PBST, membranes were imaged with an Odyssey Infrared Imager (LiCor, Model 9120) and protein band intensities were quantified with the Odyssey Software (LiCor, V3.0).

### Time-lapse video microscopy

Stable NSC-34 cells were plated on glass coverslips and induced to express either shSC or shFUS1. Twenty-four hours later, coverslips were assembled into chambers as previously described^69^ and imaged at 37 °C with BH2 (Olympus, Center Valley, PA, USA) microscopes equipped with phase-contrast optics using 10X objectives/0.3–0.32 NA. Image sequences were gathered using Retiga EX (Qimaging, Corp., Surrey, Canada) or Retiga EXi Fast (Qimaging, Corp.) cameras. Images were acquired every 3 min with C-imaging software (Hamamatsu Photonics, Hamamatsu City, Japan) and were exported as QuickTime videos using CinePak compression (Apple, Cupertino, CA, USA).

### EdU incorporation

NSC-34 cells were plated on glass coverslips at 10 000 cells per well and induced 12–16 h later to express shSC or shFUS. After 96 h of induction, cells were exposed to 10 *μ*M EdU for 30 min and processed according to manufacturer's instructions (Click-iT EdU Alexa Fluor 488 Imaging Kit, Life Technologies (Carlsbad, CA, USA), C10337). The percentage of cells positive for EdU was determined with MetaMorph V7.6.3 software using the Multi Wavelength Cell Scoring application. Briefly, immunofluorescence images were opened in MetaMorph and using the application, cells were identified by DAPI staining and scored for EdU incorporation based on a 488 nm intensity of 1000 gray levels above background. The number of cells positive for EdU was divided by the total number of cells identified by DAPI staining to determine a percentage.

### Cell cycle analysis

Following 96 h of shRNA induction in NSC-34 cells, ~2E10+6 cells were collected in 100 *μ*l PBS, fixed by the dropwise addition of 900 *μ*l of cold (−20 ºC) 95% ethanol while vortexing gently, and stored at 4 °C overnight. Following the overnight fixation, the ethanol was removed by pelleting the cells and washing with PBS. One milliliter of staining solution (900 *μ*l PBS+2 mM MgCl_2_, 50 *μ*l propidium iodide stock solution (1 mg/ml, Sigma, P4170), 50 *μ*l RNase stock solution) was then added to the cells and incubated in the dark at 37 °C for 20 min, followed by analysis within a few hours using a FACSCalibur platform (BD Biosciences, San Jose, CA, USA).

### Small-molecule high-content screen

For the high-content screen, shFUS1 NSC-34 cells were plated onto 384-well plates (Corning (Corning, NY, USA) 3712) at 500 cells per well in 40 *μ*l of culture media (described above) using a Multidrop automated liquid handler (Thermo Fisher Scientific, Waltham, MA, USA). Gas-permeable seals were used to reduce any uneven evaporation effects. After overnight incubation, 5 *μ*l doxycycline was transferred using a Biomek NX robotic liquid handler (Beckman Coulter, Indianapolis, IN, USA) into each experimental well to attain a final concentration of 1 *μ*g/ml. An equal volume of doxycycline solvent (dd H_2_O) was added to control wells. Four hours after doxycycline addition, 5 *μ*l of compounds from the Laboratory for Drug Discovery in Neurodegeneration (LDDN) library (described below) were transferred using the robotic liquid handler into experimental wells to attain a final concentration of 1 *μ*M and 0.1% DMSO. An equal volume of DMSO in media was added to control wells. Plates were incubated for 96h. Screening of compounds was performed in triplicate because of the small activity window (25%) as determined during assay development using this format.

A subset of the LDDN chemical library was used, consisting of 606 compounds approved by the FDA (Prestwick, France) and 480 purified natural products. Compounds for high-throughput screening were stored as DMSO stocks at −20 °C and assay-ready 384-well plates with 1.67 mM compound concentration in 100% DMSO were diluted in media to attain a final concentration of 1 *μ*M in 0.1% DMSO just before use.

After 96 h, cells were fixed with 4% paraformaldehyde for 20 min and washed three times with PBS (ELx405 plate washer, BioTek (Winooski, VT, USA)). The cells were labeled with 1 *μ*g/ml Hoechst stain to visualize nuclei. Images were captured on the IN Cell Analyzer 2000 (GE Healthcare, Pittsburgh, PA, USA). The entire well was imaged in four fields at 25-ms exposure time using excitation filter D360/40 and emissions filter HQ460/40 in combination with a 10X/0.45 NA objective. Image stacks were batched and analyzed using IN Cell Workstation software (GE Healthcare). For the feature extraction protocol, cells were segmented using the multi-target analysis algorithm and nuclei were segmented as defined with a minimum area of 50 *μ*m^2^ with a sensitivity setting of 70. Total nuclei per well was used as a measure of cell count. In converting this assay from a 24-well to a 384-well format, the effect of FUS knockdown on cell viability was reduced from ~40 to ~25%, perhaps because of the reduced plate format and cell plating. This small signal window was a challenge for high-throughput screening and therefore compounds were screened in triplicate.

A compound was considered a 'hit' and selected for confirmation if it rescued the shFUS1 knockdown-induced decrease in cell number by >3 S.D. from the mean of the shFUS1 knockdown control wells (shFUS1 +doxycycline) on each of the three replicate plates. Hits were confirmed via a five-point dose-response curve (0.1–30 *μ*M) using compounds that were re-ordered from the commercial suppliers. For confirmation studies, compounds were added to both shSC and shFUS1-expressing cells in the presence of doxycycline. Western blot analysis of FUS expression after 96 h of 1 *μ*M compound incubation was performed using a similar protocol to that described above.

### Quantitative proteomics

NSC-34 cells were either uninduced, or induced to express shSC or shFUS1 for 24 or 96 h (for a total of six samples), followed by lysis with RIPA buffer (Boston BioProducts, BP-115-500) supplemented with protease inhibitors (Roche, 11836170001). Protein content was measured using a standard BCA Assay (Thermo Scientific, Waltham, MA, USA) and lysates were treated according to manufacturer's instructions, with slight method alteration in the detergent removal step. Briefly, 100 *μ*g of lysate was diluted to a final concentration of 1 *μ*g/*μ*l with 100 mM triethyl ammonium bicarbonate. Disulfide bonds were reduced with 0.5 M tris(2-carboxyethyl)phosphine for 1 h at 55 °C and cysteine residues were alkylated with 375 mM iodoacetamide for 30 min at ambient temperature, protected from light. Lysates were then treated for detergent clean up using Detergent Removal Spin Columns (Thermo Scientific) according to manufacturer's instructions and digested overnight at 37 °C with Trypsin (Promega (Madison, WI, USA), sequencing grade) in a 1 : 25 (w/w) enzyme:protein digest ratio. Amine reactive TMT reagents (Thermo Scientific, 6-plex kit) were dissolved in acetonitrile and allowed to react with peptides for 1 h at ambient temperature. After 1 h, the reaction was quenched upon addition of 5% hydroxylamine hydrochloride followed by a 15-min incubation at room temperature. Following labeling, the samples were combined in an equi-volume ratio (1 : 1 : 1 : 1 : 1 : 1) and dried down before reconstitution in 5% acetonitrile, 0.1% trifluoroacetic acid for mass spectrometric analysis. The assignment of TMT tag (126–131) to each sample was randomized (www.random.org) to guard against bias. Assignments were made such that no sample had the same tag identity between three independent experiments.

Three independent experiments (biological replicates) were tested, with each experiment analyzed in technical triplicate. LC-MS/MS experiments were performed on a QExactive hybrid mass spectrometer (Thermo Scientific) equipped with a nanoAcquity UPLC (Waters, Milford, MA, USA). Briefly, approximately 1 *μ*g of labeled peptides were loaded on a 100 *μ*m i.d. fused-silica precolumn packed with 2 cm of Magic C18AQ resin (5 *μ*m, 200Å, Michrom Bioresources, Auburn, CA, USA) at a flow rate of 4.0 *μ*l/min for 4 min in 5% acetonitrile, 0.1% formic acid (FA) and eluted using a gradient at 300 nl/min onto a 75 *μ*m i.d. analytical column packed with 25 cm of Magic C18AQ resin (3 *μ*m, 100 Å, Michrom Bioresources) to a gravity-pulled tip. Separation was achieved by applying a 5–35% acetonitrile gradient in 0.1% FA over 180 min at 300 nl/min. In detail, the time program was: 0−180 min, 5−35% B; 180−181 min, 35–90% B; 181–191 min, 90% B; 191–192 min, 90-5% B; 192–210 min, 5% B; 210 min, stop. Solvent A was water with 0.1% FA and solvent B was acetonitrile with 0.1% FA. Electrospray ionization was enabled via liquid junction into a QExactive hybrid mass spectrometer (Thermo Scientific). Instrument conditions closely matched those of the ‘sensitive' conditions previously recommended.^70^ Mass spectra were acquired over *m/z* 300–1750 at 70 000 resolution (*m/z* 200) and data-dependent acquisition selected the top 12 most abundant precursor ions for MS/MS by HCD fragmentation using an isolation width of 1.2 *m/z*, normalized collision energy of 30% and a resolution of 35 000.

Mass spectra were submitted to MASCOT (Matrix Science (Boston, MA, USA), v2.4.1) and searched against a parsed *mus musculus* database (SwissProt, July 2013, containing 16 627 entries). Search specifications considered up to two missed cleavage sites, full tryptic specificity, precursor mass tolerance of 10 p.p.m. and a fragment ion tolerance of 0.05 Da. Specified fixed modifications include carbamidomethylation (Cys) and TMT6plex were considered as fixed modifications, whereas pyroglutamylation (Gln), oxidation (Met) and acetylation (N-terminus) were considered as variable modifications.

Raw files and Mascot search results were uploaded to ProteoIQ (Premier Biosoft (Palo Alto, CA, USA), v2.6.03) for quantitation based upon reporter ion signal intensity (*m/z* 126–131). The normalized reporter ion intensity was determined from the sum of reporter ions intensities across all experimental conditions and technical replicates for each peptide. Normalized ion intensities were then applied to each individual reporter ion for each peptide and weighted by the corresponding peptide precursor ion intensity. The quantitation values of each individual reporter ion from all peptides within a protein were then averaged to yield an overall protein quantitation value for each experimental condition. Quantification parameters included: reporter ion tolerance – 0.05 Da; minimum peptide length – 6 amino acids; minimum # of spectra – 2; minimum # of peptides – 2; minimum peptide probability – 0.05; minimum protein probability – 0.5 and data centroided. After protein set generation, data were further filtered for statistical significance with the following stringencies: *P*-value ≤ 0.05 and fold change ≥|0.25|.

### Quantitative real-time PCR (qRT-PCR)

RNA was harvested from cells (Bio-Rad, 732-6820) and converted to cDNA (Bio-Rad, 170-8841), according to the manufacturer's instructions. qRT-PCR was performed in technical triplicate using PrimePCR primer assays (Bio-Rad, 100-25636) and SYBR Green Supermix (Bio-Rad, 172-5121) according to manufacturer's guidelines and using the following PCR program in a CFX384 Touch Real-Time PCR Detection System (Bio-Rad): 95 °C for 2 min, 40 cycles of 95 °C for 5 s and 60 °C for 30 s, and a melt-curve of 65–95 °C (0.5 °C increments per 5 s). Quantification of differential mRNA expression was calculated by the comparative Ct method using Bio-Rad CFX Manger 3.1 software. Briefly, threshold cycle (Ct) values were normalized to the B2M reference gene Ct value to obtain ΔCt values. The ΔCt value for the control condition was subtracted from the ΔCt of the experimental condition to calculate a ΔΔCt. Fold change relative to the control samples was then calculated by 2^-(ΔΔCt)^.

## Figures and Tables

**Table 1:**
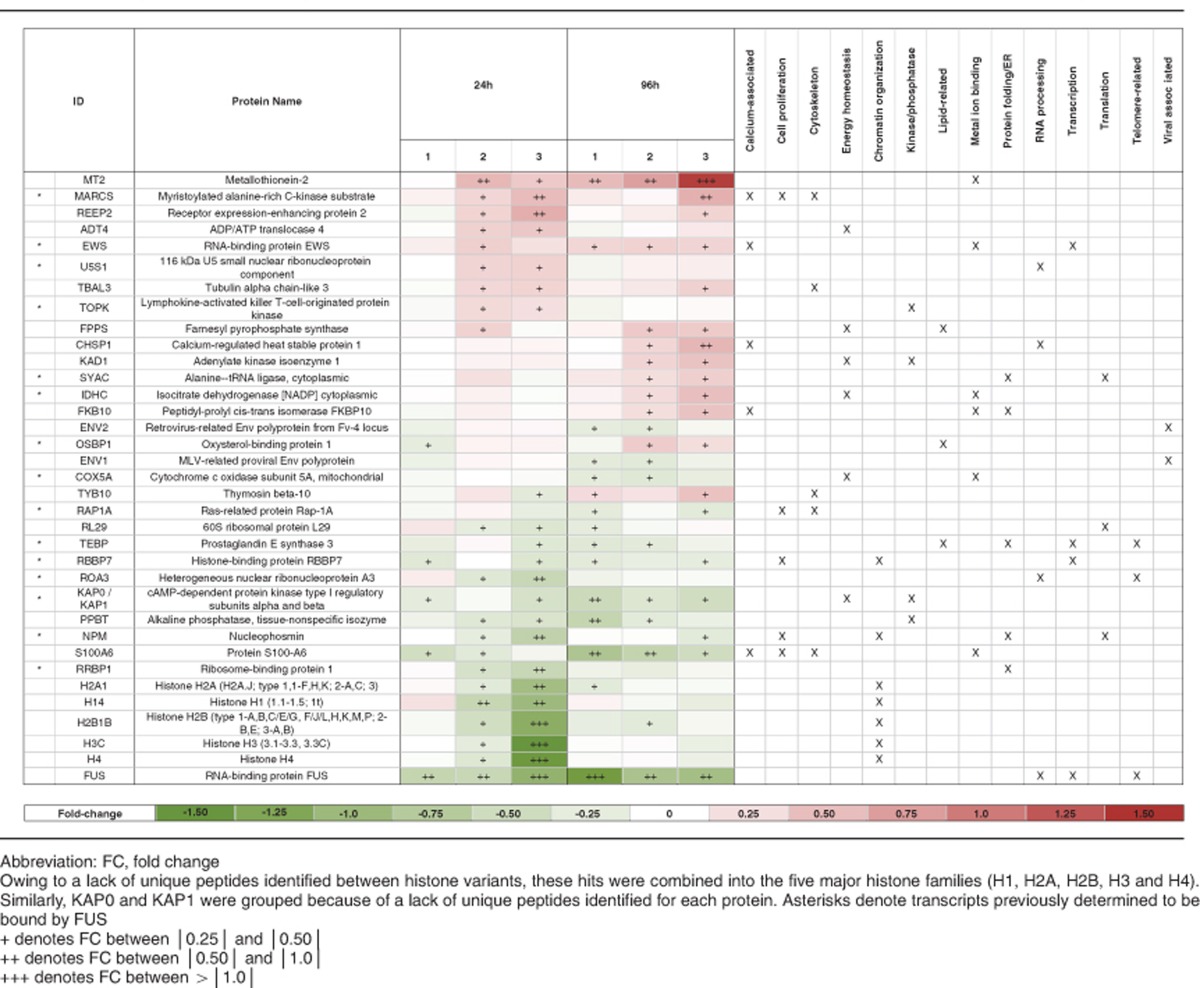
Fold change and functional categories of proteins differentially expressed after 24 or 96 h of FUS knockdown in at least two of three biological replicates

## Abbreviations

FUS: fused in sarcoma/translocated in liposarcoma
NSC-34: neuroblastoma x spinal cord hybrid 34
TMT: tandem mass tag
HEK-293T: human embryonic kidney 293T
MS/MS: tandem mass spectrometry
