# Supplementary material for: A loss of FUS/TLS function leads to impaired cellular proliferation
Source: Cell Death Dis. 2014 Dec 11;5(12):e1572–. doi: 10.1038/cddis.2014.508 (PMC4649830; doi:10.1038/cddis.2014.508)
Supplement: Supplementary Table 1 [file cddis2014508x3.doc]

| **24h** | | **Protein Name** | **shFUS/shSC** | | | | | **shSC/uninduced** | | | | **shFUS/uninduced** | | | | | | | |
| --- | --- | --- | --- | --- | --- | --- | --- | --- | --- | --- | --- | --- | --- | --- | --- | --- | --- | --- | --- |
| ***** | **MARCS** | Myristoylated alanine-rich C-kinase substrate | **0.32** | | **0.51** | | | -0.07 | | -0.30 | | 0.25 | | | | 0.21 | | | |
|  | **REEP2** | Receptor expression-enhancing protein 2 | **0.34** | | **0.62** | | | -0.14 | | -0.25 | | 0.19 | | | | 0.37 | | | |
|  | **ADT4** | ADP/ATP translocase 4 | **0.36** | | **0.43** | | | -0.24 | | -0.16 | | 0.13 | | | | 0.27 | | | |
| ***** | **U5S1** | 116 kDa U5 small nuclear ribonucleoprotein component | **0.33** | | **0.35** | | | -0.12 | | -0.17 | | 0.21 | | | | 0.18 | | | |
|  | **TBAL3** | Tubulin alpha chain-like 3 | **0.31** | | **0.34** | | | -0.19 | | -0.20 | | 0.12 | | | | 0.13 | | | |
| ***** | **TOPK** | Lymphokine-activated killer T-cell-originated protein kinase | **0.29** | | **0.25** | | | -0.11 | | -0.09 | | 0.18 | | | | 0.15 | | | |
|  | **RL29** | 60S ribosomal protein L29 | **-0.25** | | **-0.36** | | | -0.04 | | 0.17 | | -0.30 | | | | -0.19 | | | |
| ***** | **RBBP7** | Histone-binding protein RBBP7 | **-0.31** | | **-0.36** | | | 0.10 | | 0.07 | | -0.21 | | | | -0.30 | | | |
| ***** | **ROA3** | Heterogeneous nuclear ribonucleoprotein A3 | **-0.32** | | **-0.56** | | | 0.07 | | 0.42 | | -0.25 | | | | -0.14 | | | |
| ***** | **KAP0 / KAP1** | cAMP-dependent protein kinase type I regulatory subunits | **-0.26** | | **-0.42** | | | 0.12 | | 0.14 | | -0.15 | | | | -0.28 | | | |
|  | **PPBT** | Alkaline phosphatase, tissue-nonspecific isozyme | **-0.32** | | **-0.39** | | | -0.01 | | -0.09 | | -0.33 | | | | -0.30 | | | |
| ***** | **NPM** | Nucleophosmin | **-0.25** | | **-0.64** | | | 0.17 | | 0.50 | | -0.08 | | | | -0.14 | | | |
| ***** | **RRBP1** | Ribosome-binding protein 1 | **-0.42** | | **-0.56** | | | 0.04 | | 0.30 | | -0.38 | | | | -0.27 | | | |
|  | **FUS** | RNA-binding protein FUS | **-0.61** | **-0.56** | | | **-1.09** | 0.04 | 0.43 | | 0.75 | -0.57 | | -0.13 | | | | -0.35 | |
|  | | | | | | | | | | | | | | | | | | | |
|  | **MT2** | Metallothionein-2 | **0.55** | | **0.42** | | | -0.25 | | -0.39 | | 0.30 | | | | 0.03 | | | |
|  | **S100A6** | Protein S100-A6 | **-0.47** | | **-0.30** | | | 0.42 | | 0.26 | | -0.05 | | | | -0.04 | | | |
|  | **H1** | Histone H1 (t; 1.1-1.5) | **-0.59** | | **-0.98** | | | 0.50 | | 1.02 | | -0.09 | | | | 0.05 | | | |
|  | **H2A** | Histone H2A (type 1; 1-F,H,K; 2-A,C; 3; J) | **-0.29** | | **-0.94** | | | 0.56 | | 1.08 | | 0.27 | | | | 0.14 | | | |
|  | **H2B** | Histone H2B (type 1-A,B,C/E/G,F/J/L,H,K,M,P; 2-B,E; 3-A,B) | **-0.47** | | **-1.15** | | | 0.45 | | 1.00 | | -0.02 | | | | -0.15 | | | |
|  | **H3** | Histone H3 (C; 3.1-3.3) | **-0.26** | | **-1.50** | | | 0.33 | | 1.34 | | 0.07 | | | | -0.16 | | | |
|  | **H4** | Histone H4 | **-0.34** | | **-1.43** | | | 0.38 | | 1.22 | | 0.04 | | | | -0.22 | | | |
| **96h** | | **Protein Name** | **shFUS/shSC** | | | | | **shSC/uninduced** | | | | **shFUS/uninduced** | | | | | | | |
|  | **MT2** | Metallothionein-2 | **0.52** | **0.73** | | | **1.43** | -0.33 | -0.26 | | -0.94 | | 0.19 | | 0.47 | | | | 0.49 |
| ***** | **EWS** | RNA-binding protein EWS | **0.32** | **0.35** | | | **0.33** | -0.12 | -0.21 | | -0.22 | | 0.19 | | 0.14 | | | | 0.12 |
|  | **CHSP1** | Calcium-regulated heat stable protein 1 | **0.32** | | **0.56** | | | -0.14 | | -0.28 | | | 0.17 | | | | 0.29 | | |
|  | **TYB10** | Thymosin beta-10 | **0.27** | | **0.47** | | | 0.19 | | -0.07 | | | 0.46 | | | | 0.40 | | |
| ***** | **SYAC** | Alanine--tRNA ligase, cytoplasmic | **0.26** | | **0.37** | | | -0.13 | | -0.18 | | | 0.13 | | | | 0.19 | | |
| ***** | **OSBP1** | Oxysterol-binding protein 1 | **0.39** | | **0.26** | | | -0.66 | | -0.18 | | | -0.26 | | | | 0.08 | | |
| ***** | **RAP1A** | Ras-related protein Rap-1A | **-0.27** | | **-0.29** | | | 0.26 | | 0.12 | | | -0.01 | | | | -0.17 | | |
|  | **ENV2** | Retrovirus-related Env polyprotein from Fv-4 locus | **-0.25** | | **-0.28** | | | 0.02 | | 0.07 | | | -0.23 | | | | -0.20 | | |
|  | **H2B1A** | Histone H2B type 1-A | **-0.25** | | **-0.30** | | | 0.34 | | -0.09 | | | 0.09 | | | | -0.39 | | |
|  | **ENV1** | MLV-related proviral Env polyprotein | **-0.27** | | **-0.30** | | | 0.04 | | 0.09 | | | -0.23 | | | | -0.21 | | |
| ***** | **RBBP7** | Histone-binding protein RBBP7 | **-0.28** | | **-0.25** | | | 0.15 | | 0.08 | | | -0.13 | | | | -0.17 | | |
| ***** | **TEBP** | Prostaglandin E synthase 3 | **-0.31** | | **-0.28** | | | 0.21 | | 0.13 | | | -0.10 | | | | -0.15 | | |
| ***** | **COX5A** | Cytochrome c oxidase subunit 5A, mitochondrial | **-0.27** | | **-0.32** | | | 0.34 | | 0.02 | | | 0.07 | | | | -0.31 | | |
|  | **PPBT** | Alkaline phosphatase, tissue-nonspecific isozyme | **-0.61** | | **-0.29** | | | 0.09 | | 0.04 | | | -0.52 | | | | -0.25 | | |
| ***** | **KAP0 / KAP1** | cAMP-dependent protein kinase type I regulatory subunits | **-0.53** | **-0.35** | | | **-0.48** | 0.31 | -0.07 | | 0.27 | | -0.22 | | -0.42 | | | | -0.22 |
|  | **S100A6** | Protein S100-A6 | **-0.83** | **-0.62** | | | **-0.49** | 0.65 | 0.14 | | 0.34 | | -0.18 | | -0.48 | | | | -0.15 |
|  | **FUS** | RNA-binding protein FUS | **-1.39** | **-0.86** | | | **-0.93** | 0.53 | 0.53 | | 0.13 | | -0.86 | | -0.34 | | | | -0.81 |
|  | | | | | | | | | | | | | | | | | | | |
|  | **FPPS** | Farnesyl pyrophosphate synthase | **0.42** | | | **0.41** | | -0.74 | | -0.50 | | | -0.32 | | | | -0.08 | | |
| ***** | **IDHC** | Isocitrate dehydrogenase [NADP] cytoplasmic | **0.28** | | | **0.49** | | -0.48 | | -0.50 | | | -0.19 | | | | -0.02 | | |
|  | **FKB10** | Peptidyl-prolyl cis-trans isomerase FKBP10 | **0.26** | | | **0.46** | | -0.26 | | -0.34 | | | -0.01 | | | | 0.11 | | |
|  | **KAD1** | Adenylate kinase isoenzyme 1 | **0.33** | | | **0.46** | | -0.46 | | -0.29 | | | -0.13 | | | | 0.17 | | |
